# 2680. A Dangerous Comeback: Delivery Hospitalizations and Sexually Transmitted Diseases in Mississippi, 2016-2021

**DOI:** 10.1093/ofid/ofad500.2291

**Published:** 2023-11-27

**Authors:** Manuela Staneva, Thomas Dobbs

**Affiliations:** Mississippi State Department of Health, Hattiesburg, Mississippi; University of Mississippi Medical Center, Jackson, Mississippi

## Abstract

**Background:**

The United States is facing a new public health crisis: a surge in sexually transmitted diseases (STDs). The increase in STDs among pregnant patients is of special concern because these infections could lead to delivery complications and poor neonatal outcomes. To respond to this public health emergency, we examined trends and prevalence of STDs among pregnant patients admitted for deliveries in Mississippi.

**Figure:**
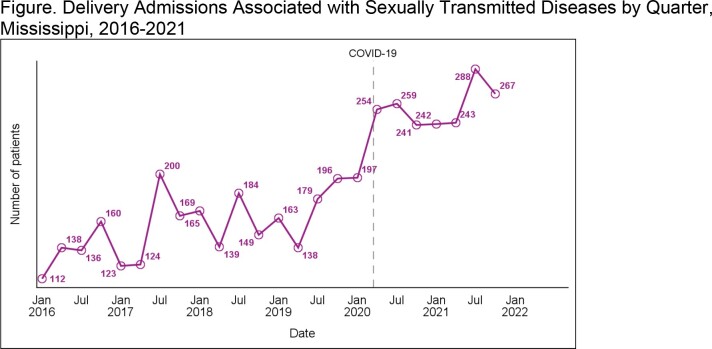

**Methods:**

This was a retrospective cross-sectional study of Mississippi’s hospital discharge data for the 2016-2021 period. This population-level data set contains demographic, clinical, and financial information from all non-federal hospitals in the state. The study included pregnant Mississippi residents (12-49 years) with a delivery admission. The unit of analysis was individual patients. We used the International Classification of Diseases-10-Clinical Modification to identify STDs among delivery admissions. We described patients' characteristics and performed trend analyses.

**Results:**

Of the 4,466 (2.2%) delivery admissions with at least one coexisting STD diagnosis, the majority involved African Americans (62.2%), Medicaid-insured (64.6%), and non-urban residents (56.9%). Herpes simplex was the most prevalent infection, accounting for 57.0% of all STD-associated delivery admissions, followed by trichomoniasis (16.0%), other/unspecified STDs (11.0%), syphilis (8.5%), chlamydia (7.9%), and gonorrhea (4.8%).

Between 2016 and 2021, STD-associated delivery admissions rose 90.9%, beginning this increase in 2017 but soaring in 2019 right before COVID-19 (Figure). Alarmingly, syphilis-associated delivery admissions demonstrated the highest increase (545.0%). Herpes simplex (134.9%) and chlamydia (69.1%) also rose significantly. Over the course of the study period, only trichomoniasis-associated and gonorrhea-associated delivery admissions remained stable.

**Conclusion:**

We identified an explosive upward trend in escalating STDs among delivery admissions in Mississippi. Especially troubling is the spike in syphilis among such patients. This study can provide public health structures with urgently needed information on the distribution and trends of STDs among Mississippi’s most vulnerable populations.

**Disclosures:**

**All Authors**: No reported disclosures

